# Rapid one-pot radiosynthesis of [*carbonyl*-^11^C]formamides from primary amines and [^11^C]CO_2_

**DOI:** 10.1186/s41181-020-00103-y

**Published:** 2020-09-01

**Authors:** Federico Luzi, Antony D. Gee, Salvatore Bongarzone

**Affiliations:** School of Imaging Sciences & Biomedical Engineering, King’s College London, 4th Floor Lambeth Wing, St Thomas’ Hospital, London, SE1 7EH UK

**Keywords:** Carbon-11, Formamides, Carbon-11 chemistry, Formylmethionine, [^11^C]CO_2_

## Abstract

**Background:**

Formamides are common motifs of biologically-active compounds (e.g. formylated peptides) and are frequently employed as intermediates to yield a number of other functional groups. A rapid, simple and reliable route to [*carbonyl*-^11^C]formamides would enable access to this important class of compounds as in vivo PET imaging agents.

**Results:**

A novel radiolabelling strategy for the synthesis of carbon-11 radiolabelled formamides ([^11^C]formamides) is presented. The reaction proceeded with the conversion of a primary amine to the corresponding [^11^C]isocyanate using cyclotron-produced [^11^C]CO_2_, a phosphazene base (2-*tert*-butylimino-2-diethylamino-1,3-dimethylperhydro-1,3,2-diazaphosphorine, BEMP) and phosphoryl chloride (POCl_3_). The [^11^C]isocyanate was subsequently reduced to [^11^C]formamide using sodium borohydride (NaBH_4_). [^11^C]Benzyl formamide was obtained with a radiochemical yield (RCY) of 80% in 15 min from end of cyclotron target bombardment and with an activity yield of 12%. This novel method was applied to the radiolabeling of aromatic and aliphatic formamides and the chemotactic amino acid [^11^C]formyl methionine (RCY = 48%).

**Conclusions:**

This study demonstrates the feasibility of ^11^C-formylation of primary amines with the primary synthon [^11^C]CO_2_. The reactivity is proportional to the nucleophilicity of the precursor amine. This novel method can be used for the production of biomolecules containing a radiolabelled formyl group.

## Background

Positron emission tomography (PET) is a non-invasive imaging technique for the in vivo detection and monitoring of normal and abnormal molecular function in health and disease (Miller et al. 2008). PET relies on the administration of radiopharmaceuticals labelled with positron-emitting radionuclides (Miller et al. 2008; Antoni 2015; Conti and Eriksson 2016). Of all the available positron-emitting radionuclides, carbon-11 (^11^C) is a valuable choice due to the ubiquity of carbon atoms in biologically-active compounds (Miller et al. 2008; Conti and Eriksson 2016) and substituting a stable carbon atom with its positron-emitting isotope maintains the chemical and biological properties of the compound (Miller et al. 2008; Rotstein et al. 2013). However, the rapid radioactive decay of ^11^C (radioactive half-life *t*_*1/2*_ = 20.4 min) requires a rapid incorporation of carbon-11 into the target molecule to avoid activity losses during the synthesis procedure (Dahl et al. 2017). A constraint of currently used carbon-11 radiolabelling strategies is the limited choice of cyclotron-produced primary synthons available for radiolabelling (Miller et al. 2008) which are either [^11^C]carbon dioxide ([^11^C]CO_2_) or [^11^C]methane ([^11^C]CH_4_) when an oxidizing or reducing environment, respectively, are used during the proton irradiation of a nitrogen gas target (Miller et al. 2008). Despite its low chemical reactivity, [^11^C]CO_2_ has been utilized as a synthon for direct incorporation of carbon-11 into radiopharmaceuticals (Miller et al. 2008; Rotstein et al. 2013; Deng et al. 2019).

Novel [^11^C]CO_2_ direct radiolabelling strategies have been developed based on the electrophilicity of [^11^C]CO_2_, significantly improving its applicability as a synthon (Rotstein et al. 2013; Dahl et al. 2017; Deng et al. 2019; Taddei and Gee 2018; Bongarzone et al. 2017; Riss et al. 2012; Krasikova et al. 2009; van der Meij et al. 2003). These strategies include the use of: *i)* highly reactive nucleophiles (e.g. Grignard reagents or organolithium compounds) to form [^11^C]carboxylic acids (Rotstein et al. 2013; Krasikova et al. 2009; van der Meij et al. 2003); and *ii)* superbases, known as CO_2_-fixation agents, for the carboxylation of boronic esters, amines and alcohols (Rotstein et al. 2013; Dahl et al. 2017; Deng et al. 2019; Taddei and Gee 2018; Bongarzone et al. 2017; Riss et al. 2012). Superbases, such as 1,8-diazabicyclo[5.4.0]undec-7-ene (DBU) and 2-tertbutylimino-2-diethylamino-1,3-dimethyl-perhydro-1,3,2- diazaphosphorine (BEMP) (Dahl et al. 2017; Deng et al. 2019; Taddei and Gee 2018; Bongarzone et al. 2017), are capable of increasing [^11^C]CO_2_ solubility and reactivity by creating labile bonds with [^11^C]CO_2_ and have enabled the rapid and reliable synthesis of [^11^C]carboxylic acids (Riss et al. 2012), [^11^C]amides (Bongarzone et al. 2017; Aubert et al. 1997), [^11^C]ureas (Downey et al. 2018; Haji Dheere et al. 2013), [^11^C]isocyanates (Wilson et al. 2011), [^11^C]carbonates (Haji Dheere et al. 2018).

To date, direct formamide labelling is not accessible by current ^11^C-synthesis methods although the formamidic motif is found in several biologically-active molecules, such as the vitamin B1 analogue octotiamine, the thiamine analogue fursultiamine (Seddighi et al. 2015), and the chemotactic peptide formyl methionine (Schiffmann et al. 1975). Moreover, the formamidic group is a versatile intermediate due to its high chemical reactivity and can be used in electrophilic aromatic substitution to form aryl aldehydes (Downie et al. 1993) or to perform ring-closure reactions (Horkka et al. 2019; Schou and Halldin 2012). It is also a valuable reagent in the synthesis of isocyanides (Guchhait et al. 2013), quinolines (Jackson and Meth-Cohn 1995) and formamidines (Han and Cai 1997).

The availability of a simple and robust ^11^C-formamide radiolabelling method would enable access to formamide-containing PET imaging agents as well as carbon-11 labelled formamides as radiosynthetic intermediates.

**Scheme 1:**
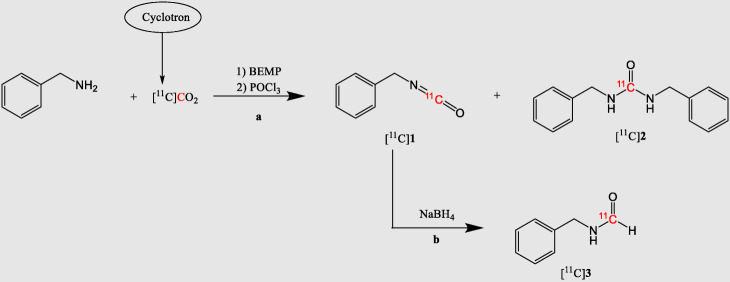
Proposed reaction scheme for the one-pot synthesis of [^11^C]formamides. Reaction conditions: **a** benzylamine (4.7 μmol, 1 equiv.), BEMP (3.7 equiv.), diglyme (75 μL), [^11^C]CO_2_, 0–20 °C, 2 min, followed by POCl_3_ (11.5 equiv.) in diglyme (75 μL), 0–20 °C, 2 min. **b** NaBH_4_ (5–15 equiv.), diglyme (50 μL), 0–60 °C, 2–15 min

**Scheme 2:**
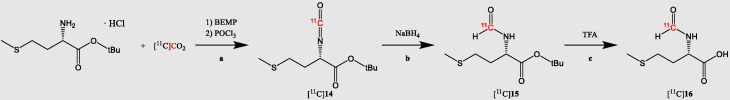
Radiolabelling of [*carbonyl*-^11^C] formyl methionine *t*Bu ester ([^11^C]**15**) and subsequent deprotection to form [*carbonyl*-^11^C] formyl methionine ([^11^C]**16**). Reaction conditions: **a** methionine *t*Bu ester (4.7 μmol, 1 equiv.), BEMP (34.6 μmol, 7.4 equiv.), diglyme (75 μL), [^11^C]CO_2_, 0 °C, 2 min, then POCl_3_ (54.05 μmol, 11.5 equiv.) in diglyme (75 μL), 0 °C, 2 min. **b** NaBH_4_ (2.67 mg, 15 equiv.), diglyme (50 μL), 0 °C, 10 min. **c** TFA (200 μL), 20 °C, 2 min

Herein, we report a rapid one-pot radiosynthesis of [^11^C]formamides from primary amines and cyclotron-produced [^11^C]CO_2_ (Scheme 1) using [^11^C]CO_2_-fixation chemistry strategies. In the presence of BEMP as superbase and a primary amine, the reaction proceeds via the initial formation of a [^11^C]isocyanate intermediate as described by Wilson et al. (Wilson et al. 2011). [^11^C]CO_2_ is trapped in a solution of benzylamine and BEMP, initially forming the corresponding [*carbonyl*-^11^C]carbamate on the aminic function (Wilson et al. 2011). The addition of a superbase serves to deprotonate the primary amine (pKa BEMP = 27.6, pKa benzylamine = 8.82), making it more reactive towards the delivered [^11^C]CO_2_ and helping the trapping of activity in the reaction vial. The subsequent addition of phosphorus(V) oxychloride (POCl_3_) dehydrates the [^11^C]carbamate, yielding the [^11^C]isocyanate intermediate ([^11^C]**1**, Scheme 1) (Wilson et al. 2011) which is subsequently reduced by an excess of sodium borohydride (NaBH_4_), yielding the desired [^11^C]formamide derivative [^11^C]**3** (Scheme 1). Aliphatic and aromatic amines were also tested to investigate the applicability of the method on different chemotypes. Furthermore, the radiolabelling of the chemotactic aminoacid [*carbonyl*-^11^C]formyl methionine ([^11^C]**16**, Scheme 2) (Schiffmann et al. 1975) is reported.

## Results and discussion

Initial experiments focused on studying the formation of [^11^C]**1** following the method described by Wilson et al. (2011). By combining benzylamine (4.7 μmol, 1 equiv.), BEMP (3.7 equiv.), [^11^C]CO_2_ from the cyclotron target and POCl_3_ (11.5 equiv.) in diglyme (150 μL) at 20 °C for 4 min, [^11^C]**1** was yielded in modest radiochemical yields (RCY = 63%) (Determined by radio-HPLC analysis of the crude product n.d.) together with the ^11^C-labelled symmetric urea as a byproduct ([^11^C]**2**, RCY = 31% Scheme 1). The cyclotron-produced [^11^C]CO_2_ was quantitatively trapped in the reaction mixture (trapping efficiency (TE) > 95%) (28). Trapping efficiency (TE) of cyclotron-produced [^11^C]CO_2_ into the reaction vial was calculated by dividing the activity of the reaction vial by the total activity delivered by the cyclotron (reaction vial + ascarite).

In order to improve the RCY of [^11^C]**1**, we opted to decrease the temperature of the reaction - as lowering the temperature might reduce the reactivity of [^11^C]**1** towards the unreacted primary amine in solution (hence decreasing the formation of [^11^C]**2**). Indeed, decreasing the temperature from 20 to 0 °C significantly increased the RCY of [^11^C]**1** from 63% to 81%.

Encouraged by the increased RCY of [^11^C]**1**, we focused on its reduction to [*carbonyl*-^11^C]benzyl formamide ([^11^C]**3**, Scheme 1, Table 1). As observed in previous non-radioactive work, the reduction of isocyanates (1 equiv.) is achieved using a high excess of lithium aluminium hydride (LiAlH_4_, 15 equiv.) (Finholt et al. 1953) or sodium borohydride (NaBH_4_, 15 equiv.) (Ellzey and Mack 1963) at high temperatures (> 160 °C). The reduction step was developed starting from the latter method as the milder reducing agent NaBH_4_ would allow broader substrate scope in the method applicability. When 15 equivalents of NaBH_4_ were used at 150 °C for 15 min, high amounts of the undesired [^11^C]**2** (47%) and a polar unknown radioactive by-product (53%) were formed instead of [^11^C]**3** (entry 1, Table 1). [^11^C]**2** is not affected by these conditions and remains unchanged. The failure of the reduction might be due to the harsh conditions applied (15 equiv. of reducing agent, high temperature and long reaction time), thus milder conditions were tested by a concomitant decrease of the equivalents of NaBH_4_ (10 equiv.), temperature (20 °C) and reaction time (10 min). As a result, the desired [^11^C]**3** was obtained in good yield (RCY = 33%, entry 2, Table 1).

**Table 1:** Reduction of [^11^C] benzyl isocyanate to [^11^C] benzylformamide ([^11^C]**1**)^a^

Entry	NaBH_**4**_ Equiv.	Temp (°C)	Time (min)	RCY of [^**11**^C]3 (%)	RCY of [^**11**^C]2 (%)	RCY of a unknown radioactive by-product (%)	RCY of [^**11**^C]1 (%)
**1**^b^	15	150	15	0	47	53	0
**2**	10	20	10	33 ± 6	35 ± 8	23 ± 5	11 ± 1
**3**	5	20	10	2 ± 1	16 ± 4	3 ± 1	77 ± 3
**4**	15	20	10	57 ± 4	16 ± 3	17 ± 5	0
**5**	15	60	10	42 ± 4	38 ± 4	28 ± 8	0
**6**	15	0	10	80 ± 4	9 ± 4	6 ± 3	0
**7**	15	0	15	79 ± 6	10 ± 2	8 ± 2	0
**8**	15	0	5	60 ± 3	15 ± 4	8 ± 4	8 ± 4
**9**	15	0	2	60 ± 2	13 ± 4	9 ± 1	14 ± 2

^a^Reaction conditions for all entries: [^11^C]CO_2_ was bubbled at 0 °C for 2 min in a solution of benzylamine (4.7 μmol, 1 equiv.) and BEMP (3.7 equiv.) in diglyme (75 μL). At end of delivery (EOD, 2 min) a solution of POCl_3_ (11.5 equiv.) in diglyme (75 μL) was added in the same vial, allowing the reaction for 2 min at 0 °C. A solution of NaBH_4_ (0.89–2.67 mg, 5–15 equiv.) in diglyme (50 μL) was then added. The reduction occurred at 0–60 °C for 2–15 min. Trapping efficiency (TE) > 95% for all entries. *n* = 3. RCY (non-isolated) is the percentage of product radioactivity divided by total radioactivity observed in an analytical HPLC chromatogram

^b^*n* = 1

With the aim to increase the RCY of [^11^C]**3**, a further optimization study was performed by varying a single parameter (equivalents of reducing agent, temperature, reduction time) per experiment (entries 3–9, Table 1). Lowering the equivalents of NaBH_4_ from 10 to 5 had a detrimental effect on the RCY of [^11^C]**3** (33% in entry 2 versus 2% in entry 3, Table 1) which can be explained by the lower availability of hydride ions in solution to accomplish the reduction and is consistent with the high amount of unreacted [^11^C]**1** found in the mixture (77%, entry 3, Table 1). On the other hand, increasing the amount of NaBH_4_ from 10 to 15 equivalents showed a modest increase in [^11^C]**3** RCY from 33% to 57% (entry 2 versus entry 4, Table 1).

Next, we studied the effect of the temperature on the RCY of [^11^C]**3**. Increasing the temperature from 20 to 60 °C lowered the formation of [^11^C]**3** from 57% to 42% (entry 4 versus entry 5, Table 1). A further increase in temperature to 150 °C did not yield [^11^C]**3** (entry 1, Table 1). Lowering the temperature to 0 °C, instead, increased the RCY of [^11^C]**3** from 57% to 80% (entry 4 versus entry 6, Table 1). Hence, [^11^C]formamide formation is temperature dependent and favoured at low temperatures whereas higher temperatures promote the formation of the undesired [^11^C]**2** (9% at 0 °C, entry 6, 16% at 20 °C, entry 4, 38% at 60 °C, entry 5, 47% at 150 °C, entry 1, Table 1).

The influence of the reaction time on the RCY of [^11^C]**3** was also explored by performing the reduction with NaBH_4_ for 2, 5 and 15 min. When the reaction was allowed to proceed for 15 min, no significant change on the RCY of [^11^C]**3** was observed (79% versus 80%, entry 7 versus entry 6, Table 1). Decreasing the reduction time from 10 to 5 or 2 min still resulted in good yields of [^11^C]**3** (60% and 60% at 5 or 2 min, respectively, entries 8 and 9, Table 1). After a reaction time of either 5 or 2 min, however, the reaction did not reach completion with the unreacted [^11^C]**1** still being present in the mixture (14% and 8%, respectively, entries 8 and 9, Table 1).

**Fig. 1:**
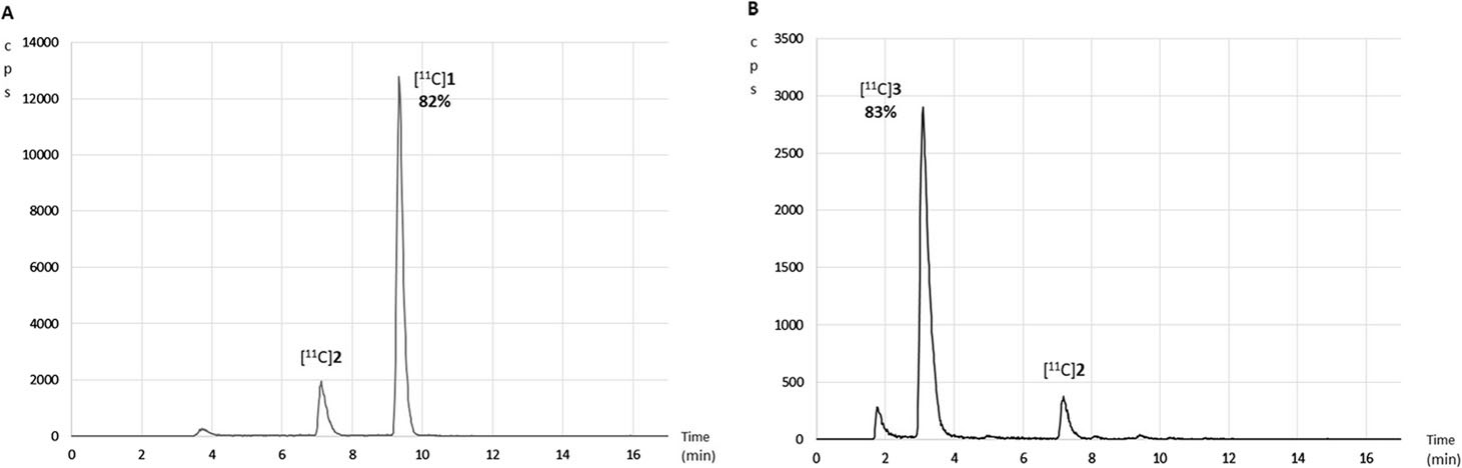
Radioactive traces of the analytical HPLC evaluation (entry 6, Table 1) **a** after [^11^C]**1** synthesis; **b** after [^11^C]**3** synthesis

In summary, this optimisation process allowed us to produce [^11^C]**3** from benzyl amine and [^11^C]CO_2_ with good RCY (80%, entry 6, Table 1, Fig. 1) and high TE (> 95%) in 15 min from the release of [^11^C]CO_2_ from the cyclotron target and an activity yield of 12% and a molar activity (A_m_) of 5 ± 2 $\frac{GBq}{\mu mol}$ (decay-corrected at end of bombardment (EOB) with an initial delivery of 300 MBq of [^11^C]CO_2_).^1^

To study the scope of the reaction, the aforementioned reaction conditions were subsequently applied to a number of amines (**4**, **6**, **8** and **10**) to produce the corresponding [*carbonyl*-^11^C] formamides ([^11^C]**5**, [^11^C]**7**, [^11^C]**9** and [^11^C]**11**, Table 2). 
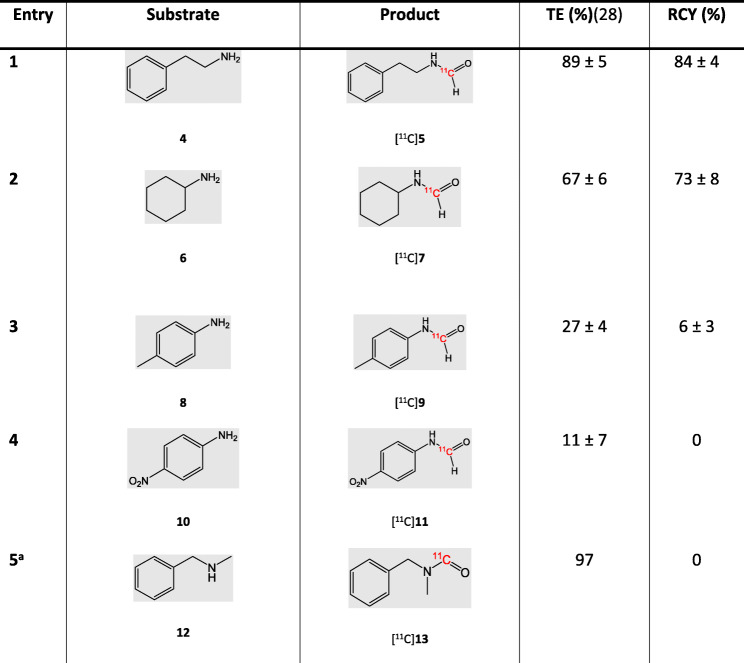

**Table 2:**
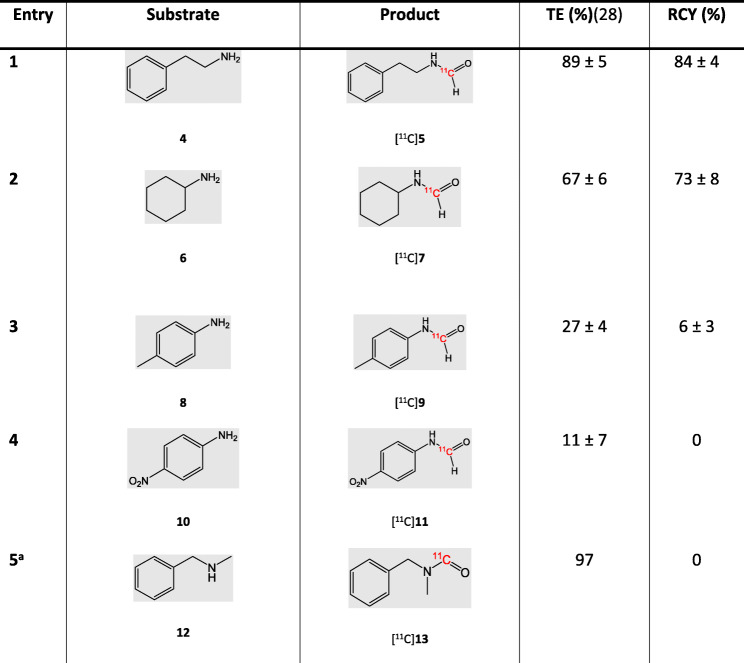
Conversion of aliphatic and aromatic amines to their corresponding [*carbonyl*-^11^C] formamide derivatives

Reaction conditions for all entries: [^11^C]CO_2_ was bubbled at 0 °C for 2 min in a solution of amine (4.7 μmol, 1 equiv.) and BEMP (3.7 equiv.) in diglyme (75 μL). At end of delivery (EOD, 2 min) a solution of POCl_3_ (11.5 equiv.) in diglyme (75 μL) was added in the same vial, allowing the reaction for 2 min at 0 °C. A solution of NaBH_4_ (2.67 mg, 15 equiv.) in diglyme (50 μL) was then added. The reduction occurred at 0 °C for 10 min. n = 3. RCY (non-isolated) is the percentage of product radioactivity divided by total radioactivity observed in an analytical HPLC chromatogram.

^a^*n* = 1

Initially, the effect of the spacer between the amine group and the phenyl ring was investigated by adding one extra carbon on the alkyl chain of benzylamine. Correspondingly, 2-phenethylamine (**4**) was used as starting material. Whilst the TE was slightly lower than that observed with [^11^C]**3** ([^11^C]**3** > 95% and [^11^C]**5** = 89 ± 5%, entry 1, Table 2), the product [^11^C]*N*-(phenethyl) formamide ([^11^C]**5**) was achieved with a higher RCY ([^11^C]**3** = 80 ± 4% and [^11^C]**5** = 84 ± 4%), which might be explained by the higher nucleophilicity of **4** (pK_a_ benzylamine = 8.82 versus pK_a_ **4** = 9.73).

The reactivity of a hindered primary amine was also studied by employing cyclohexylamine (**6**) as a substrate. As expected, both TE and RCY of [^11^C]*N*-cyclohexylformamide ([^11^C]**7**) were lower than other amines tested (TE = 67%; RCY = 73%, entry 2, Table 2) due to the lower nucleophilicity of the substrate and the steric hindrance that partly impede the reaction.

Aromatic amines were subsequently tested by using activated (*p*-toluidine, **8**, pK_a_ = 5.10) and deactivated (*p*-nitroaniline, **10**, pK_a_ = 1.01) aromatic rings. Testing these two amines would also reveal the effect of different substitution patterns on the reactivity of the substrate. When **8** was used as a substrate, the TE was significantly lower than previous entries (27%, entry 3, Table 2) and the respective [^11^C]*p*-formotoluidide ([^11^C]**9**) was obtained with low RCY (6%, entry 3, Table 2). Using deactivated aromatic rings such as **10** further lowered the TE (11%, entry 4) and did not yield any product. These results are in line with previous findings where aromatic amines had shown low [^11^C]CO_2_ trapping and reactivity (Wilson et al. 2011) and highlight the relevance of aromatic ring substituents, with activated rings being more reactive than deactivated aromatic systems. These experiments also highlight the importance of the basicity of the starting amine: precursors with higher basicity will bind the delivered [^11^C]CO_2_ with higher TE, resulting in higher RCY (Figure SI5, Supplementary Information). The radiolabelling of a secondary amine as a negative control was also tested (entry 5, Table 2) to confirm the reaction mechanism. The use of a secondary amine would indeed prevent the formation of the [^11^C]isocyanate intermediate, thus impeding the proceeding of the reaction. When *N*-benzylmethylamine (**12**) was used as a substrate, the product [^11^C]*N*-benzyl-*N*-methylformamide ([^11^C]**13**) was not formed.

Next, this novel radiolabelling strategy was applied to the synthesis of the chemotactic peptide [*carbonyl*-^11^C] formyl methionine (Scheme 2) by using the hydrochloric form of the *tert*-butyl (*t*Bu) ester of methionine as starting material. The cyclotron-produced [^11^C]CO_2_ was delivered at 0 °C in the reaction vial containing methionine *t*Bu-ester and BEMP. The subsequent addition of POCl_3_ and the reaction for 2 min at 0 °C formed the [^11^C]isocyanate analogue ([^11^C]**14**, Scheme 2). [^11^C]**14** was then reduced with an excess of NaBH_4_ for 10 min to yield [*carbonyl*-^11^C]*t*Bu-formylmethioninate ([^11^C]**15**, Scheme 2) in good RCY (57%). The *t*Bu protecting group was subsequently removed by adding TFA (200 μL) at r.t. for 2 min, forming the desired [*carbonyl*-^11^C]formyl methionine ([^11^C]**16**, Scheme 2) with a RCY of 48% within 18 min from EOB.

## Conclusions

In summary, this proof-of-concept study demonstrates the feasibility of direct ^11^C-formylation of aromatic and aliphatic primary amines using the primary synthon [^11^C]CO_2_. The reaction proceeds via the formation of a [^11^C]isocyanate intermediate via [^11^C]CO_2_ fixation chemistry. The [^11^C]isocyanate was subsequently reduced to the [^11^C]formamidic analogue. The total processing time was 15 min (EOB to end of synthesis (EOS)), with [^11^C]**3** produced in high RCY (80%) and high TE (> 95%). To confirm the applicability of the developed method, an array of aliphatic and aromatic amines was tested. When aliphatic amines were used, the respective [^11^C]formamides were produced in high yields (RCYs = 74–83%), whereas aromatic amines showed little-to-no reactivity (RCYs = 0–6%). Thus, the reactivity is related to the nucleophilicity of the amine. The radiolabelling of a secondary amine as negative control was attempted, as well, resulting in no formation of the desired product and confirming the reaction mechanism. Furthermore, the radiolabelling of the biologically-relevant compound [*carbonyl*-^11^C]formyl methionine was successfully attempted with a RCY of 48% (Determined by radio-HPLC analysis of the crude product n.d.) in 18 min from EOB to EOS. This method could be applied to the radiolabelling of an array of formylated radiopharmaceuticals including the chemotactic peptide [^11^C]*N*-formylmethionine-leucyl-phenylalanine ([^11^C]fMLP) to study inflammation and [^11^C]formoterol for the imaging of β2 adrenergic receptors in pulmonary diseases.

## Methods

### Carbon-11 chemistry

*N*-benzylamine (99%), 2-tertbutylimino-2-diethylamino-1,3-dimethyl-perhydro-1,3,2-diazaphosphorine (BEMP), phosphorus(V) oxychloride (POCl_3_), sodium borohydride (NaBH_4_), benzyl isocyanate, *N*-formyl methionine, di-tert-butyl dicarbonate and anhydrous diethylene glycol dimethyl ether (diglyme, 99.5%) were purchased from Sigma-Aldrich. Tert-butyl alcohol and *N*-benzyl formamide (99%) was purchased from Alfa Aesar. The purchased *N*-benzyl formamide and benzyl isocyanate were used as HPLC reference. *N*-formyl methionine was purchased from Sigma-Aldrich, L-methionine t-butyl ester hydrochloride was purchased from ChemCruz.

The reactions were performed in oven-dried v-shaped vials (KX Microwave Vials, 5 mL) sealed with crimp caps (Fisherbrand, centre hole with 3.0 mm PTFE seal aluminum silver 20 mm, part #10132712). All gas transfer lines were fabricated from PTFE tubing (length: 10–30 cm, O.D.: 0.79 × 0.4 in., I.D.: 1/32 × 0.16 in.).

[^11^C]CO_2_ was produced using a Siemens RDS112 cyclotron by the 11 MeV proton bombardment of nitrogen (+ 0.5% O_2_) gas via the ^14^N(p,α)^11^C reaction. The cyclotron-produced [^11^C]CO_2_ was bubbled in a stream of helium gas with a flow rate of 60 mL/min post target depressurisation directly into an oven-dried v-shaped vial without further gas processing (time from end of bombardment (EOB) to end of delivery (EOD) = 1 min and 50 s).

The reactions were performed on a semi-automatic Eckert & Ziegler (E&Z) Modular-Lab radiochemistry synthesis module.

HPLC analysis was performed on an Agilent 1200 system equipped with a UV detector (λ = 214/254 nm) and a β^+^-flow detector coupled in series.

A P_2_O_5_ trap and a one-way valve (BRAUN, normally closed backcheck valve, part #415062) were placed before the vial. An ascarite® trap consisting of a cartridge (Supelco, Empty Reversible SPE Tube, non-fluorous polypropylene volume 1 mL) filled with ascarite (Sigma-Aldrich, 1310-73-2) was placed after the Vial to trap any unreacted [^11^C]CO_2_. A waste bag (Tedlar® gas sampling bag, 3.8 L capacity with septum) was placed at the outlet to prevent any gaseous emission.

The reaction vial containing *N*-benzylamine and BEMP in anhydrous diglyme was initially placed in the reactor and the temperature was set at 0 °C.

**Fig. 2:**
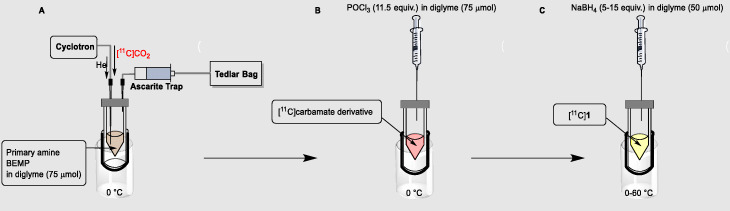
Graphical representation of the set-up used for the reaction. **a** The reaction vial is connected directly to the cyclotron target to allow the delivery of [^11^C]CO_2_. An ascarite trap and waste bag are placed after the reaction vial to trap any [^11^C]CO_2_ not fixed in the reaction vial. **b** The reaction vial is disconnected from the cyclotron target line and from the waste bag to avoid loss of [^11^C]CO_2_ during the reaction steps. Then POCl_3_ is added. **c** NaBH_4_ addition

A cyclotron beam current of 5 μA was maintained for a bombardment time of 1 min for all reaction optimization experiments producing ~ 300 MBq of carbon-11. The cyclotron-produced [^11^C]CO_2_ was bubbled into the reaction vial in a stream of helium gas with a flow rate of 50–60 mL/min post target depressurisation. An ascarite trap and a waste bag were attached to the vial via a vent needle to avoid activity loss in the environment (Fig. 2).

At end of delivery (EOD), a solution of POCl_3_ (11.5 equiv.) in anhydrous diglyme was added in the reaction vial. The magnetic agitation was turned on and the reaction occurred for 2 min. A solution of NaBH_4_ (5–15 equiv.) in anhydrous diglyme was subsequently added to the main reaction vial, leaving it to react for 2–15 min (Fig. 2).

The reaction was then quenched with 300 μL of mobile phase composed of water and acetonitrile (H_2_O:ACN) 60:40. To avoid overpressure of gases inside the vial due to free hydrogen production, a waste bag was attached to the vial via a vent needle.

The trapping of cyclotron-produced [^11^C]CO_2_ into the reaction vial was calculated by dividing the activity of the reaction vial by the total activity delivered from the cyclotron (reaction vial + ascarite).

An aliquot of the crude was injected in the radio-HPCL in order to determine the RCY.

### Molar activity calculation for [^11^C]3

Eight samples of **3** at different concentrations (0.2–0.00039 mM) were analysed by HPLC to obtain a calibration curve of the peak area (mAU*s) versus μmol/mL (Figure SI4). The peak areas of **3** were averaged and plotted in function of the corresponding μmol/mL.

An aliquot of purified [^11^C]**3** (20 μL) was analysed by analytical radioHPLC and the UV peak corresponding to **3** was integrated. The area of the UV peak was used to determine the μmol/mL of the associated ^12^C-carrier content for [^11^C]**3** from the equation of the calibration curve. The molar activity (A_m_) of [^11^C]**3** was calculated to be 4.57 ± 1.99 GBq/μmol (*n* = 3) decay-corrected at EOB with an initial delivery of 300 MBq.

### Synthesis of *tert butyl*-formylmethioninate (13)

**Scheme 3:**
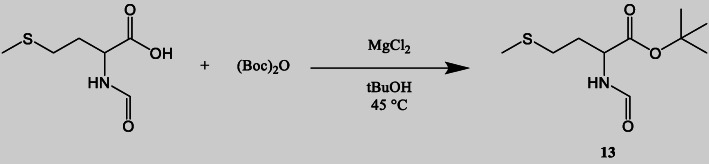
Synthesis of **13**. Reaction conditions: *N*-formyl methionine (1 equiv., 564 μmol), anhydrous magnesium chloride (0.1 equiv.), di-tert-butyl dicarbonate (0.7 equiv.), tert-butyl alcohol (2 equiv.), 45 °C, 48 h

*N*-formyl methionine (1 equiv., 564 μmol) and tert-butyl alcohol (2 equiv., 1128 μmol) were mixed in an oven-dried v-shaped vial. The reaction vial was then put into an oil bath at a temperature of 45 °C. Subsequently, di-tert-butyl dicarbonate (0.7 equiv., 394.8 μmol) and magnesium chloride (0.1 equiv., 56.4 μmol) were added in the reaction mixture. The vial was crimped and the reaction stirred for 48 h at 45 °C (Scheme 3).

After, the crimped vial was cooled to room temperature and the reaction mixture was quenched with 10 mL of water. Three aliquots of 10 mL of dichloromethane were used to extract the product. The organic fractions were then washed with a saturated solution of sodium bicarbonate, dried on anhydrous magnesium sulphate and evaporated in vacuo.

The compound was characterized via reverse-phase HPLC using an analytical column (Phenomenex Luna, 5 μm C18, 150 × 4.6 mm) with a flow rate of 1 mL/min. The gradient was isocratic until 2:30 min (ACN:H_2_O, 20:80), linear between 2:30–10 min (up to ACN:H_2_O, 95:5), isocratic between 10 and 13 min (ACN:H_2_O, 95:5) and linear between 13 and 14 min to return to initial conditions (ACN:H_2_O, 20:80) which were kept isocratic until the end of the run (17 min). The detected retention time was t_r_ = 8 min and 40 s (Figure SI5B).

^1^H-NMR was performed on Bruker AVANCE III HD 400 MHz.

^1^H-NMR of **13** (CDCl_3_): δ 1.20 (s, 9H), 1.42 (s, 3H), 2.00–2.09 (m, J = 8.67 Hz, 2H), 2.40–2.50 (m, J = 9.25, 2H), 3.52 (s, 1H), 7.20 (s, 1H).

^13^C-NMR of **13** (CDCl_3_): δ 14.81, 26.53, 32.04, 52.95, 81.28, 163.46, 170.16.

MASS (m/z): 234.10.

## Supplementary information

**Additional file 1.**

## Data Availability

All data generated or analysed during this study are included in this published article (and its supplementary information files).

## Abbreviations

^11^C: Carbon-11
[^11^C]CH_4_: Carbon-11 labelled methane
[^11^C]CO_2_: Carbon-11 labelled carbon dioxide
A_m_: Molar activity
BEMP: 2-*tert*-butylimino-2-diethylamino-1,3-dimethylperhydro-1,3,2-diazaphosphorine
DBU: 1,8-diazabicyclo [5.4.0] undec-7-ene
EOB: End of bombardment
EOS: End of synthesis
NaBH_4_: Sodium borohydride
PET: Positron emission tomography
POCl_3_: Phosphorus(V)oxychloride
RCY: Radiochemical yield
TE: Trapping efficiency

## Footnotes

1 This work describes a method development study using short, low current, cyclotron irradiations where obtaining high A_m_ were not the main focus. However, the associated carrier content of compound [^11^C]**3** was in the range of 12–14 nmol in 3 mL. Assuming that the stable ^12^C carrier content would be in the same range for a standard clinical [^11^C]CO_2_ production, it is estimated that molar activities of 290–570 $\frac{GBq}{\mu mol}$ would be obtained (decay-corrected at EOB).
