# Supplementary material for: Rapid one-pot radiosynthesis of [carbonyl-11C]formamides from primary amines and [11C]CO2
Source: EJNMMI Radiopharm Chem. 2020 Sep 1;5:20. doi: 10.1186/s41181-020-00103-y (PMC7462944; doi:10.1186/s41181-020-00103-y)
Supplement: Supplementary file 1 — Additional file 1. [file 41181_2020_103_MOESM1_ESM.docx]

**Rapid, One-Pot Radiosynthesis of [*carbonyl*-^11^C]Formamides from Primary Amines and [^11^C]CO_2_.**

Federico Luzi, Antony D. Gee, Salvatore Bongarzone.

School of Imaging Sciences & Biomedical Engineering, 4^th^ floor Lambeth Wing, St Thomas' Hospital, King's College London, London SE1 7EH, United Kingdom

***Supplementary Information***

**Table of content:**

|  | **Topic** | **Page** |
| --- | --- | --- |
| **1.** | **Radio-HPLC traces for [^11^C]1 and [^11^C]3** | **1** |
| **2.** | **Molar activity calculation for [^11^C]3** | **4** |
| **3.** | **Relationship Between pKa and Reactivity of the Used Substrates** | **4** |
| **4.** | **Radio-HPLC traces for [^11^C]15 and [^11^C]16** | **4** |
| **5.** | **References** | **7** |

**1. Radio-HPLC traces for [^11^C]1 and [^11^C]3**

An analytical reverse-phase column (Phenomenex Luna, 5 μm C18, 150 x 4.6 mm) was used with a flow rate of 1 mL/min to analyse the reaction crude. The gradient was isocratic until 2:30 min (ACN:H_2_O, 40:60), linear between 2:30–10 min (to ACN:H_2_O, 95:5), isocratic between 10–13 min (ACN:H_2_O, 95:5) and linear between 13–14 min to return to initial conditions (ACN:H_2_O, 40:60) which were kept isocratic until the end of the run (17 min). [^11^C]Benzyl isocyanate ([^11^C]**1**) was identified by co-injection with the unlabelled reference standard (**Figure SI1**). The retention time of [^11^C]**1** is 9 minutes and 20 seconds.

**
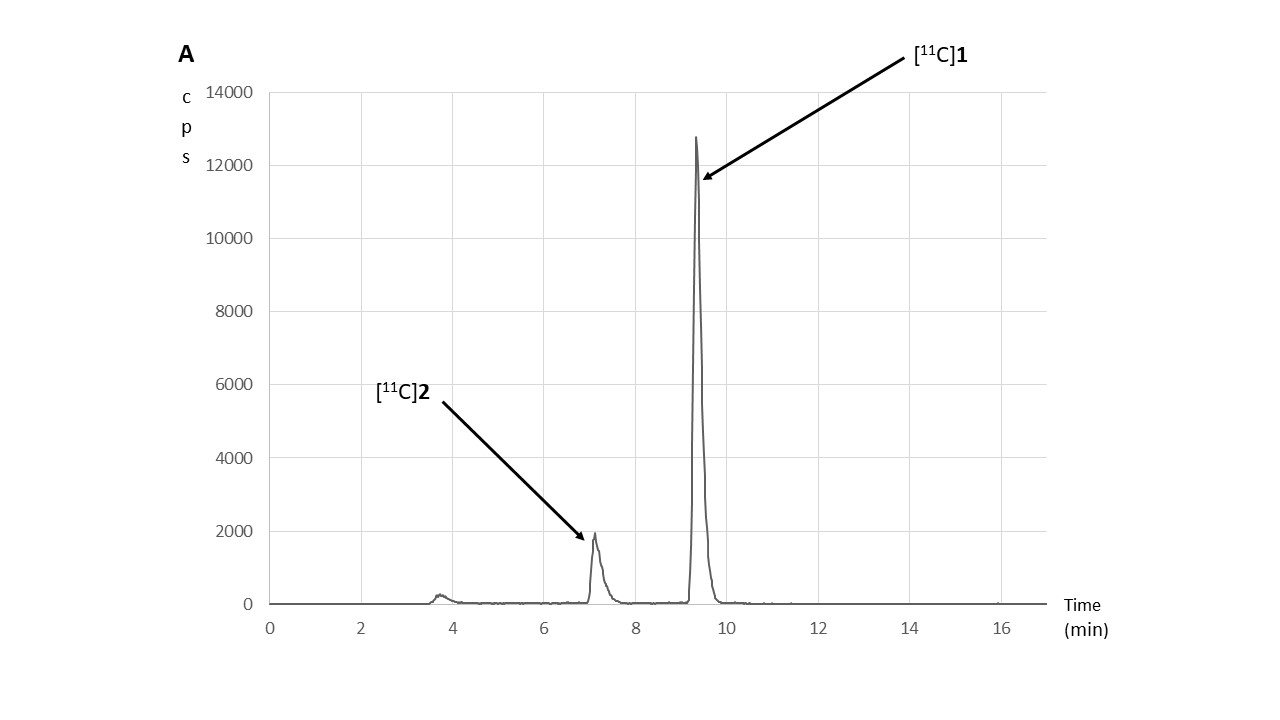
**


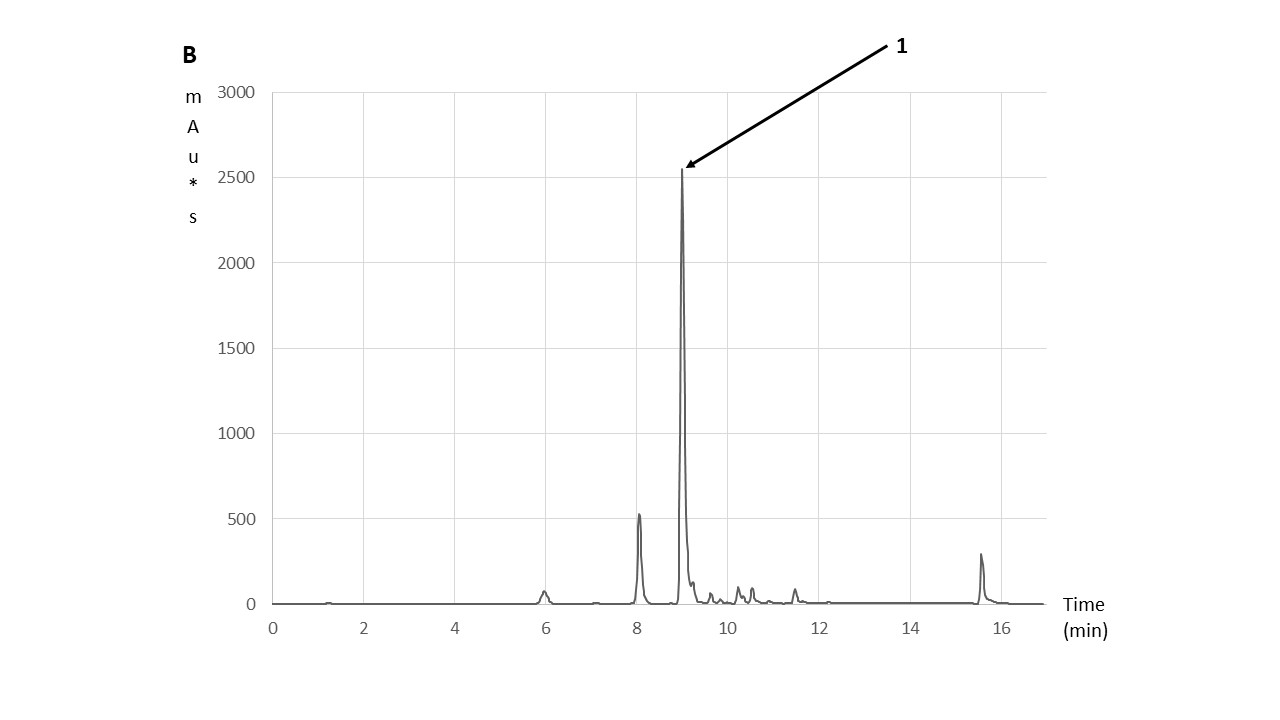


**Figure SI1.** A. Radioactive trace of the analytical HPLC evaluation of reaction crude containing [^11^C]**1**. **B.** UV trace of the analytical HPLC after the co-injection of the crude containing [^11^C]**1** and the unlabelled reference compound **1**.

[^11^C]Benzyl formamide ([^11^C]**3**) was purified by semi-preparative reverse-phase HPLC. A semi-preparative Phenomenex Luna, 5 μm C18, 250 x 10 mm was used with a flow rate of 2.5 mL/min to purify [^11^C]Benzyl formamide ([^11^C]**3**) from the reaction mixture. This HPLC method exploited the same gradient that was used for the analytical evaluation of the reaction crude. Identification of all radioactive products was confirmed by co-elution with the corresponding non-radioactive references. The retention time of [^11^C]**3** was 8 minutes and 15 seconds (**Figure SI2**). Radiochemical purity (RCP) was assessed by analysing the isolated peak associated to [^11^C]**3** via analytical radioHPLC (**Figure SI3A**). Identity of the radiolabelled product was confirmed by co-injection with the unlabelled reference standard (**Figure SI3B**).


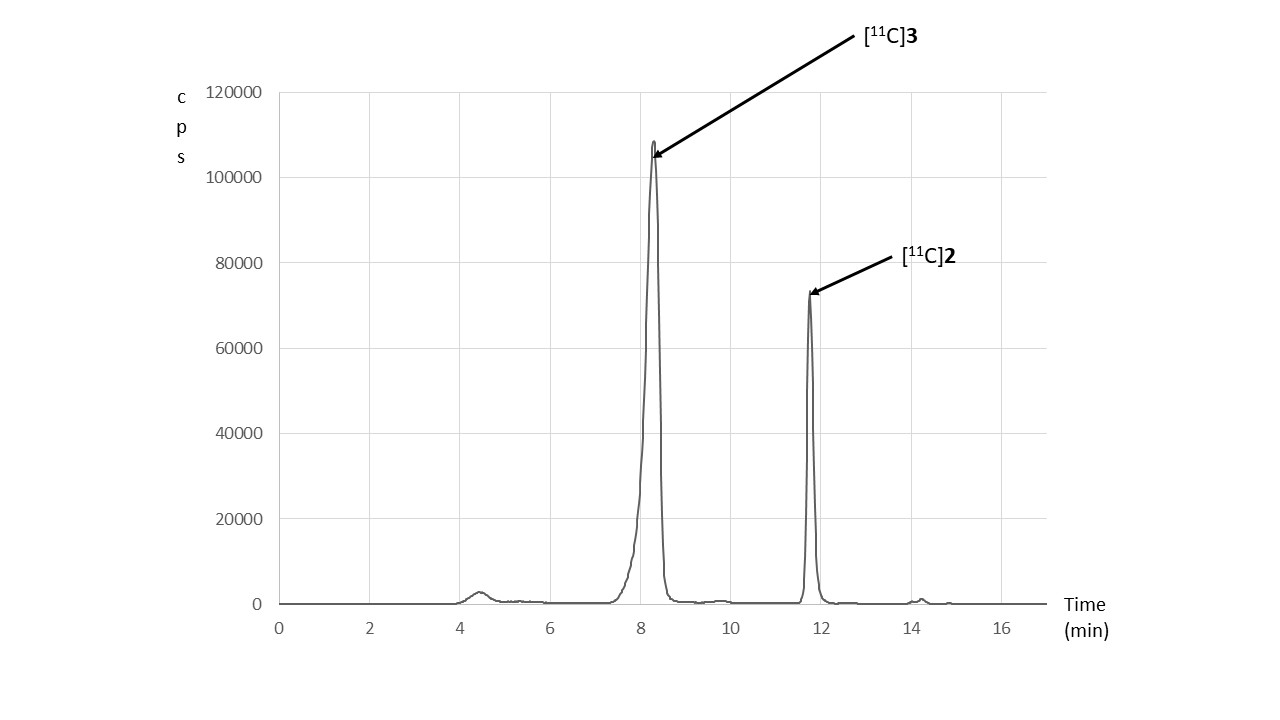


**Figure SI2.** Radioactive trace of the semi-preparative HPLC purification of the reaction crude to obtain [^11^C]**3**.


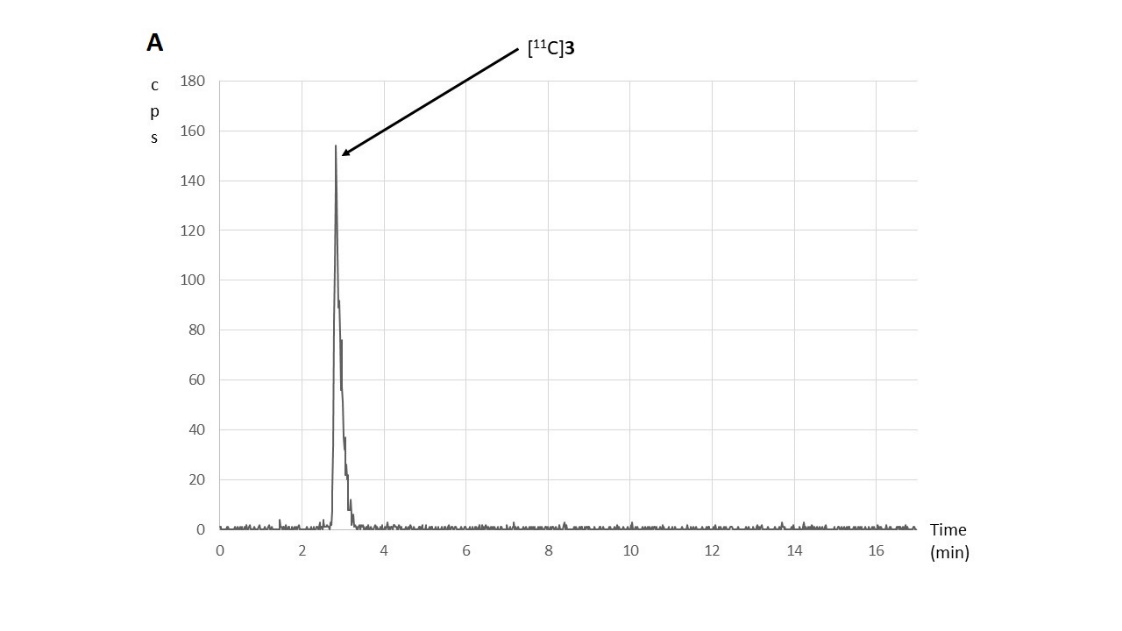


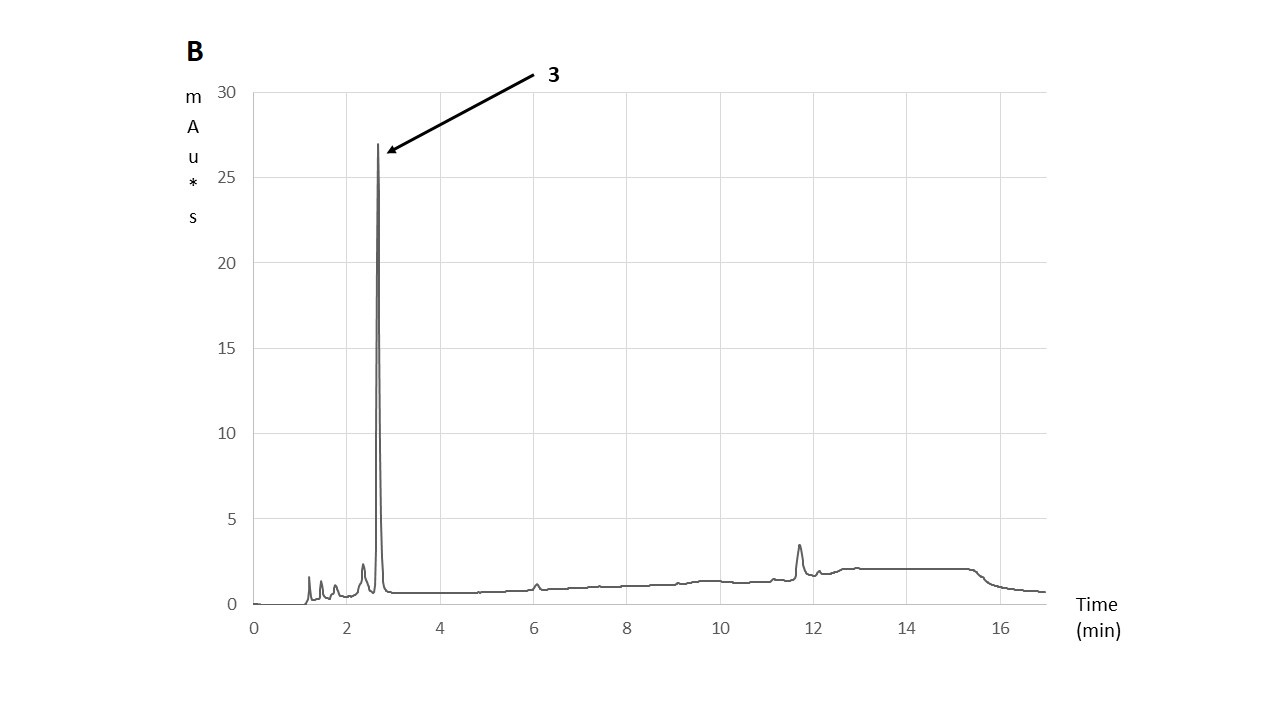


**Figure SI3.** **A.** Radioactive trace of the analytical HPLC evaluation of purified [^11^C]**3**. **B.** UV trace of the analytical HPLC after the co-injection of the purified [^11^C]**3** and the reference compound **3**.

**2. Molar activity calculation for [^11^C]3**


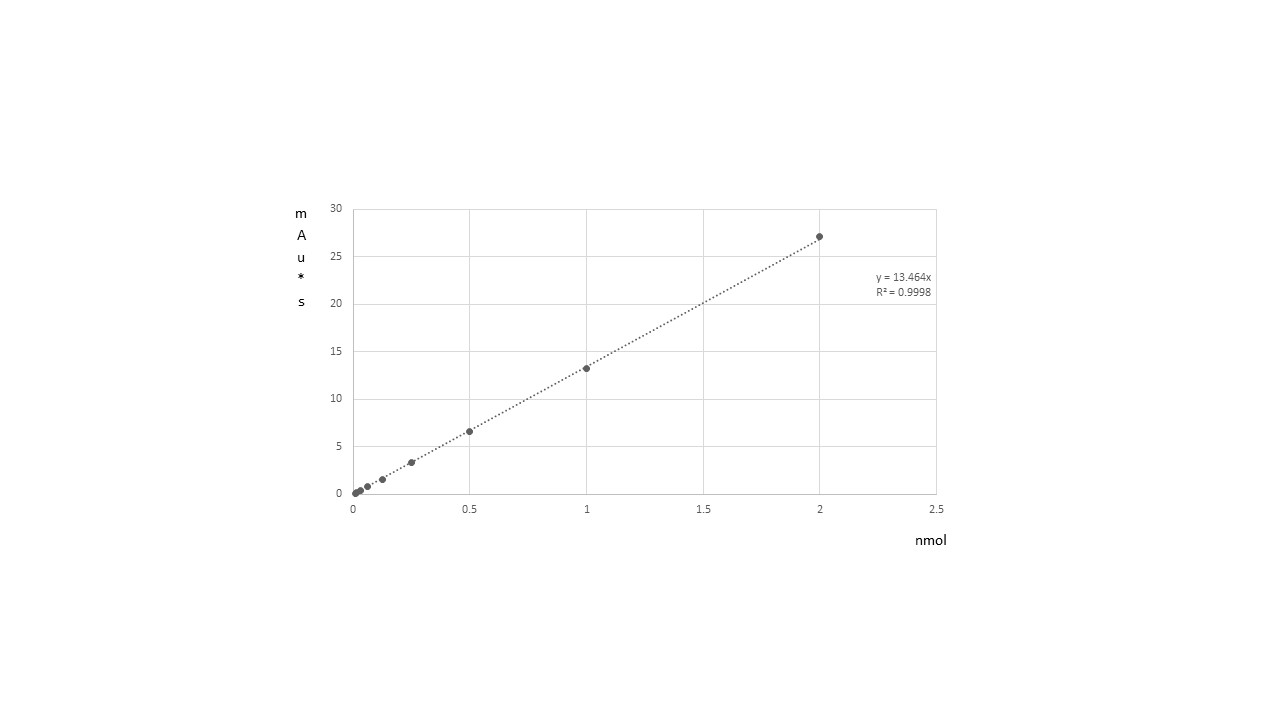


**Figure SI4.** Calibration curve for the molar activity of [^11^C]**3**.

**3. Relationship Between pKa and Reactivity of the Used Substrates**


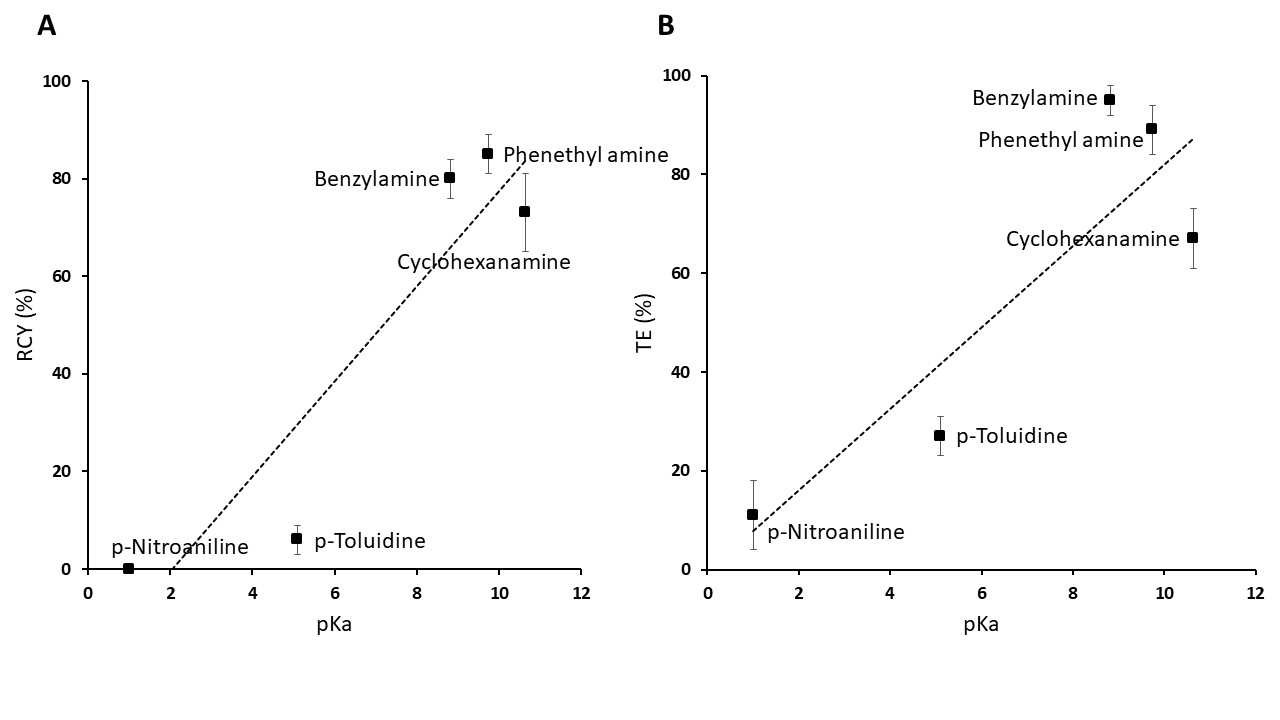


**Figure SI5.** Graphical representation of the relationship of pKa *versus* RCY (**A**) and TE (**B**).

**3. Radio-HPLC traces for [^11^C]13 and [^11^C]14**

[*carbonyl*-^11^C]*t*Bu-formylmethioninate ([^11^C]**13**) was identified by co-injection of the HPLC reference compound (**15**, **Figure SI6**). A Phenomenex Luna, 5 μm C18, 150 x 4.6 mm was used as column. The gradient was isocratic until 2:30 min (ACN:H_2_O, 20:80), linear between 2:30–10 min (to ACN:H_2_O, 95:5), isocratic between 10–13 min (ACN:H_2_O, 95:5) and linear between 13–14 min to return to initial conditions (ACN:H_2_O, 20:80) which was kept isocratic until the end of the run (17 min). The retention time of [^11^C]**15** is 8 minutes and 54 seconds.


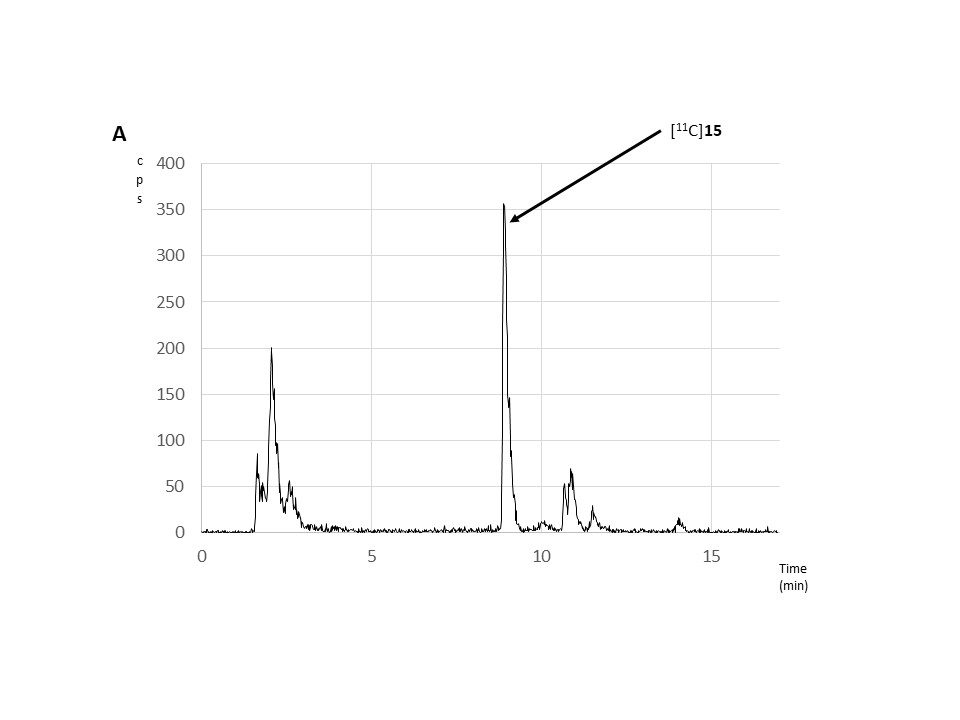

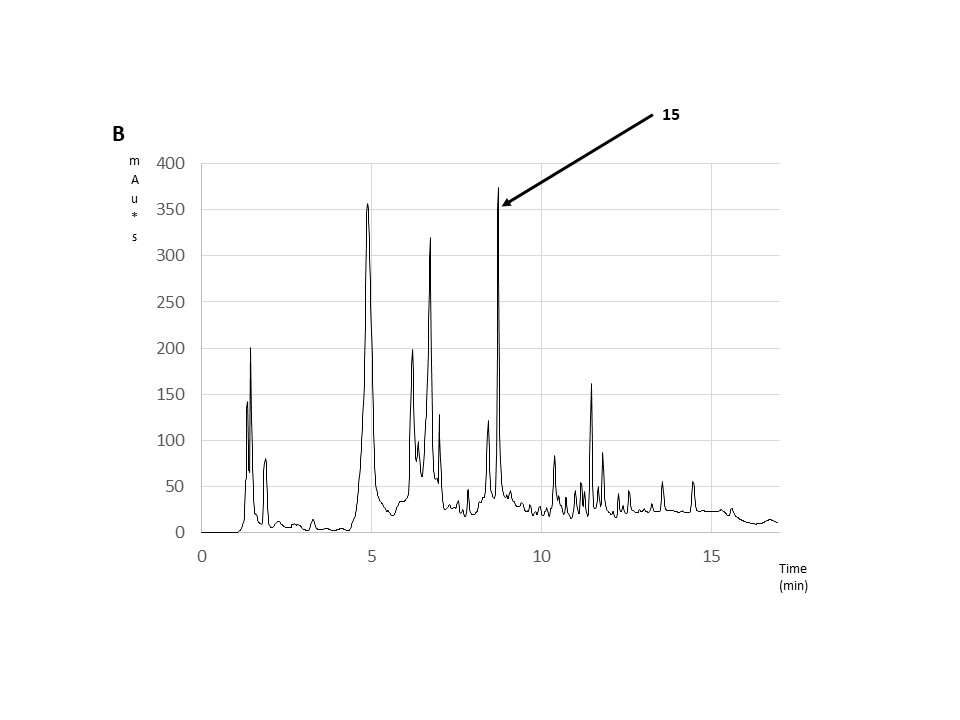


**Figure SI6.** **A.** Radioactive trace of the analytical HPLC evaluation of the reaction crude to obtain [^11^C]**15**. **B.** UV trace of the analytical HPLC of the reaction crude co-injected with the reference compound **15**.

[*Carbonyl*-^11^C]Formyl methionine ([^11^C]**16**) was identified by analytical HPLC using a Phenomenex Luna, 5 μm C18, 150 x 4.6 mm as column. The gradient was isocratic until 2:30 min (ACN:H_2_O, 5:95), linear between 2:30–10 min (to ACN:H_2_O, 80:20), isocratic between 10–13 min (ACN:H_2_O, 80:20) and linear between 13–14 min to return to initial conditions (ACN:H_2_O, 5:95) which were kept isocratic until the end of the run (17 min). The retention time of [^11^C]**16** is 3 minutes and 15 seconds (**Figure SI7A**). The identity was confirmed by comparing the retention time with an HPLC reference compound **16** (**Figure SI7B**). Although the enantiomeric purity of [^11^C]**16** has not been checked, the use of NaBH_4_ and BEMP with harsh conditions, in organic chemistry, has shown not to cause racemization of enantiomerically pure starting materials.^1-3^


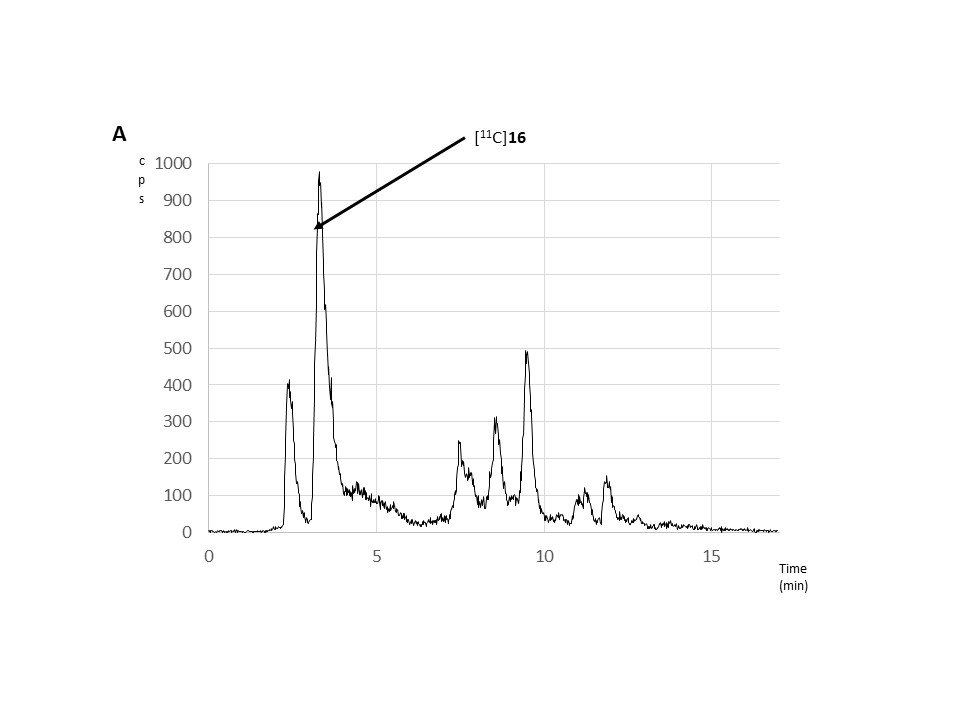

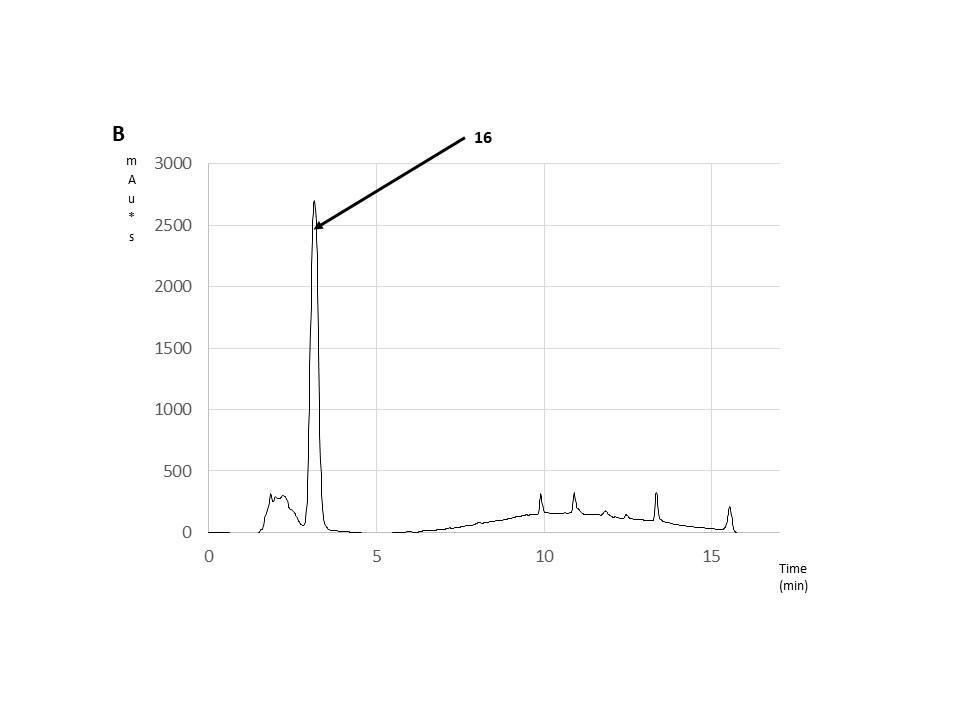


**Figure SI7.** **A.** Radioactive trace of the analytical HPLC evaluation of the reaction crude to obtain [^11^C]**16**. **B.** UV trace of the analytical HPLC of the reaction mixture co-injected with the reference compound **16**.

**4. References:**

(1) McKennon, M. J.; Meyers, A. I.; Drauz, K.; Schwarm, M. *The Journal of Organic Chemistry* **1993**, *58*, 3568.

(2) O'Donnell, M. J.; Drew, M. D.; Pottorf, R. S.; Scott, W. L. *Journal of Combinatorial Chemistry* **2000**, *2*, 172.

(3) O'Donnell, M. J.; Zhou, C.; Scott, W. L. *Journal of the American Chemical Society* **1996**, *118*, 6070.
